# Development and evaluation of an AI model for dental implant type detection: A comparison of diagnostic accuracy between a deep learning model and dental professionals

**DOI:** 10.1111/jopr.70064

**Published:** 2025-11-26

**Authors:** Walaa Magdy Ahmed, Amr Ahmed Azhari, Abdulrahman Almufti, Zainab Majed Alsadah, Amr Fawzy Abdelhamid Ahmed, Anas Lahiq, Khaled Ahmed Fawaz

**Affiliations:** ^1^ Department of Restorative Dentistry, Faculty of Dentistry King Abdulaziz University Jeddah Saudi Arabia; ^2^ Master Prosthodontic Program, Faculty of Dentistry Riyadh Elm University Riyadh Saudi Arabia; ^3^ Dental Department East Jeddah General Hospital, Ministry of Health Jeddah Saudi Arabia; ^4^ Department Artificial Intelligence Tachyhealth Company Lewes Delaware USA; ^5^ Department of Prosthodontics Aseer Dental Specialized Center Abha Saudi Arabia; ^6^ Department of Orthopedic Surgery, Faculty of Medicine Cairo University Cairo Egypt

**Keywords:** Deep Learning Model, dental implant, dental professionals, YOLOv12

## Abstract

**Purpose:**

To develop a deep‐learning system for identifying five dental implant brands from periapical radiographs and compare its diagnostic accuracy with dental professionals and evaluate successive You Only Look Once (YOLO) architectures (v7–v12) to justify model selection.

**Materials and Methods:**

A dataset of 5851 periapical radiographs was compiled and divided into training, validation, and test partitions (80/10/10). After filtering to five brands (Adin, Dentium, Noris, OSSTEM, and Straumann), YOLO‐based object detection models (versions 7–12) were trained and tested under identical conditions. The YOLOv12x model was adopted for final evaluation based on its optimal balance of accuracy and inference speed. Human performance was assessed using 100 held‐out test images (20 per brand) via a multiple‐choice web survey distributed to six clinician groups. Diagnostic metrics included mean average precision at IoU 0.50 (mAP@50), mAP@50–95, precision, recall, and accuracy.

**Results:**

The model achieved an mAP@50 of 0.989 (98.9%), an mAP@50–95 of 0.900 (90.0%), a precision of 0.969 (96.9%), and a recall of 0.977 (97.7%) across brands. Across YOLO generations, performance improved from mAP@50–95 = 0.817 (YOLOv7) to 0.905 (YOLOv12x). Fifty‐two clinicians completed 5,200 image evaluations; the model significantly outperformed all clinician subgroups (one‐way ANOVA with Tukey HSD, *p* < 0.001). Transformer‐based DF‐DETR achieved mAP@50–95 = 0.878, confirming YOLOv12x's superior efficiency–accuracy trade‐off.

**Conclusions:**

A high‐performing model identified implant brands on periapical radiographs and outperformed clinicians across experience levels. Comparative analysis across YOLO architectures validated its measurable advantage in accuracy and speed. Lack of external validation and dataset imbalance are important limitations; future work will include external, multisite data and human–AI workflow evaluation.

Artificial intelligence (AI) is no longer a mere prospect in dentistry but has become an established reality.^1^, ^2^ It employs technology that enables machines to mimic human cognitive functions.^3^ AI has gained increasing popularity worldwide, particularly within the field of prosthodontics. As materials and clinical workflows continue to transition to digital formats,^4^, ^5^, ^6^ the adoption of digital technologies has improved prosthodontic practice. These technologies leverage computational capabilities, data processing, and extensive datasets to develop customized algorithms, resulting in improved clinical outcomes and more accurate diagnostics.

Prosthodontics relies on dental implants that are supported or retained by both removable and fixed dental prostheses following implant placement surgery. Increased global mobility has led to variations in implant system sizes and types. Patients seeking information about their implants require a method to identify the implant system independent of the dentist's expertise. The accurate identification of dental implant systems is crucial in prosthodontic practice, ensuring proper treatment planning, component selection, and maintenance. In implantology, AI helps identify different implant systems and determine implant location, size, and length through the analysis of radiographic images.^7^, ^8^ AI is a potent tool that utilizes machine‐based algorithms to analyze and classify complex data. Convolutional neural networks (CNNs) and deep learning (DL), which represent cutting‐edge models in AI, have demonstrated remarkable diagnostic and predictive performance in radiology and pathology studies.^9^ Although these techniques hold significant potential to support dentistry and clinical decision‐making, the collection of large‐scale data from clinics and electronic health systems remains challenging.^10^

Among such models, the You Only Look Once (YOLO) algorithm—a real‐time object identification technique—has gained popularity due to its high detection speed, accuracy, and generalization capability.^11^ The YOLOv5 model, initially introduced in 2020, has undergone continuous updates, whereas the YOLOv7 model, released in July 2022, has demonstrated superior performance compared with other real‐time detection models. The YOLOv12 model, developed in February 2025, represents the latest advancement in this series.

Takahashi et al. used a dataset of 1282 panoramic radiographs with implants to detect dental implants using the Yolov3 algorithm and TensorFlow and Keras deep‐learning libraries.^12^ Lee et al. also evaluated the effectiveness of a deep CNN algorithm in identifying and categorizing implant systems using 5,380 periapical (PA) radiographic images and 5,390 panoramic images from three dental implant systems: OSSTEM Implant, Dentium, and Straumann SLActive.^13^

Kong et al. evaluated a DL model using panoramic radiographic images.^14^ They retrieved 14,037 implant images and categorized them into 10 classes. Three‐fold cross‐validation showed YOLOv7 had higher mean average precision scores. Benakatti et al. evaluated the effectiveness of machine learning (ML) in classifying implant systems based on shape and design using panoramic radiographic images.^15^ ML has been applied to the classification of dental implant systems using large datasets of panoramic radiographic images. Algorithms such as support vector machines, k‐nearest neighbors, XGBoost, and logistic regression were employed in the analysis. An average accuracy of 0.67 was achieved, with logistic regression demonstrating the highest performance among the trained classifiers. A study by Lee et al. found that using an automated DL algorithm, dental professionals' classification accuracy of dental implants significantly improved from 63.13% without the algorithm to 78.88%, emphasizing its importance.^16^

Most previous studies relied on panoramic radiographic images to classify implants, utilized earlier versions of AI software, and did not compare the model performance with that of dental implant specialists. Moreover, the accurate selection of appropriate dental implant systems is crucial for repairing or replacing existing implants when relevant information is unavailable. However, research on methods that enable the precise identification of implant type, size, and length remains limited. This study evaluated a state‐of‐the‐art object‐detection model (YOLOv12x) for implant brand classification from periapical radiographs and compared its diagnostic accuracy against multiple clinician subgroups. The hypothesis was that the model's accuracy would exceed human accuracy.

## MATERIALS AND METHODS

### Dataset

The study followed the Standards for Reporting of Diagnostic Accuracy Studies (STARD) and the Checklist for Artificial Intelligence in Medical Imaging (CLAIM). This study was conducted in collaboration with the Faculty of Dentistry, King Abdulaziz University, Jeddah, Saudi Arabia, and the Faculty of Dentistry, Riyadh Elm University, Riyadh, Saudi Arabia. The study adhered to ethical considerations and Institutional Review Board guidelines set by both institutions, ensuring compliance with all relevant policies. Data anonymization was performed to maintain patient confidentiality. Ethical approval was granted prior to data collection, and informed consent was obtained from all participants. This study was approved by the Research Ethics Committee of the Faculty of Dentistry, King Abdulaziz University, Jeddah, Saudi Arabia (33‐03‐25) on April 29, 2025, and the Faculty of Dentistry, Riyadh Elm University, Riyadh, Saudi Arabia (FPGRP/2024/871/1261/1134) on March 12, 2025.

### Data preparation

The methodology was divided into two phases. Phase I involved the development of the AI model and its performance assessment. Phase II involved a comparative analysis between the AI model and dental professionals. For phase I, a study power of 80% and a significance level of 0.05 (5%) were selected. A dataset of 6,092 PA radiographic images was compiled from two primary sources: the AI Implant Dataset (Proprietary Straumann data), Version v1 (released on March 2, 2025), which contains 1425 images of the Straumann implant brand (Switzerland), collected from January 2019 to February 2025, and the Implant System Detection Dataset of Multiple Implant Brands, Version v7 (released on August 8, 2024), which comprises 4667 images. The selected implant brands included Adin, DIOnavi, Dentium, MIS, Noris, Nobel Biocare, and OSSTEM.

PA radiographic images were obtained in JPEG format. Images from the AI Implant Dataset were resized (stretched) to 640 × 640 pixels, whereas those from the Implant System Detection Dataset were standardized to 800 × 800 with letter‐boxing to preserve aspect ratio prior to training/evaluation. The initial class distribution showed a significant imbalance, with high representation for OSSTEM (4122), Straumann (1669), and Adin (1581); moderate representation for Noris (1111) and Dentium (896); and low representation for MIS (411), DIOnavi (45), and Nobel (15). To optimize model performance, classes with insufficient samples (DIOnavi, MIS, and Nobel) were excluded. The filtered dataset comprised five classes (Adin, Dentium, Noris, OSSTEM, and Straumann). Each radiograph was manually annotated using the RoboFlow software (https://app.roboflow.com/). Images were resized, normalized, and augmented using rotation, flipping, and contrast adjustment techniques to enhance model performance.

### Automated model building

To clarify the AI model, this study employed YOLOv12x, a state‐of‐the‐art DL object detection model. YOLOv12x was chosen for its real‐time detection capabilities, advanced architecture, improved accuracy over previous YOLO versions, and effective performance in medical image classification. YOLOv12x was selected for its strong balance of accuracy and inference speed on object shapes comparable to implant fixtures in periapical x‐rays; in preliminary pilot experiments it outperformed prior YOLO baselines on our data while remaining production‐feasible (Table 1). Model training was conducted using the PyTorch framework. The Adam optimizer with a learning rate of 0.001 was used as the optimization algorithm, and categorical cross‐entropy loss was assessed. Data splits were performed; a compiled dataset of 5851 periapical radiographs was split into training/validation/test partitions (80/10/10). After dataset filtering, the final structure consisted of 4680 images with corresponding labels for training, 584 images for validation, and 587 images for testing.

**TABLE 1:** Comparative evaluation of YOLO architectures.

Model	Precision	Recall	mAP@50	mAP@50–95	Key notes
YOLOv7	0.976	0.932	0.974	0.817	Baseline convolutional; lower recall
YOLOv8	0.983	0.983	0.993	0.915	Decoupled head; strong overall accuracy
YOLOv9	0.979	0.965	0.986	0.885	Unified transformer backbone
YOLOv10	0.974	0.963	0.987	0.890	Gradient‐flow optimization
YOLOv11	0.983	0.977	0.993	0.915	Highest fine‐grained localization
**YOLOv12x (optimized)**	**0.976**	**0.967**	**0.990**	**0.905**	Balanced accuracy + speed; adopted in study

Abbreviation: mAP, mean average precision.

Model training was performed using the following parameters: a pre‐trained YOLOv12x base model, 800 × 800 pixel input resolution, a batch size of 16, 160 epochs (with early stopping after 10 epochs without improvement), the default YOLOv12x optimizer (auto), initial and final learning rates set at 0.01, and data augmentation comprising 50% random horizontal flipping, 10% copy‐paste, and 40% random erasing.

### Metrics for accuracy comparison

Classification accuracy, precision, recall, F1‐score, confusion matrices, and ROC analysis were used as evaluation metrics. The following formulas were applied: accuracy = (TP + TN)/(TP + FP + FN + TN); precision = TP/(TP + FP); recall = TP/(TP + FN); specificity = TN/(TN + FP); F1 = 2 × (precision × recall) / (precision + recall), where TP represents true positive, TN represents true negative, FP represents false positive, and FN represents false negative.

### Comparing the performance of YOLOv12x with that of dental professionals

Phase II involved a comparative analysis between the developed AI model and dental professionals for implant classification. Sixteen core dental professionals (4 board‐certified periodontists, 4 board‐certified prosthodontists, 4 periodontics residents, 4 prosthodontics/restorative residents) were selected based on the effect size reported in a previous study. The study included two groups: Group 1, AI alone (YOLOv12 model); and Group 2, dental professionals. A short standardized calibration was done (2–3 annotated example brands with answer keys) prior to performing the survey to ensure a comparable baseline. A self‐administered questionnaire containing 100 PA radiographic images (20 per brand), randomly selected from the test dataset, was distributed using Google Forms. The accuracy, sensitivity, specificity, and inter‐rater agreement (measured by Cohen's kappa statistic) were calculated to compare the AI model's performance with that of dental professionals. These images were excluded from model training. A total of 52 human participants were categorized into six clinical groups and evaluated 100 radiographic images using a multiple‐choice questionnaire. The participants included general dentists (with 0–5 years and more than 5 years of experience), residents (periodontics and prosthodontics/restorative), and consultants (periodontal and prosthodontic/restorative). Each participant selected one brand or the option “I don't know” for each image. Predictors made by both the AI model and the dental professionals were recorded as either “true” or “false.” A “true” result indicated that the selected implant type matched the actual implant system shown in the radiograph. A “false” result indicated a mismatch. The independent variable in this study was the method of detection (developed model, or dental implant professionals).

### Statistical analysis

Primary comparison among human groups used one‐way ANOVA with Tukey HSD; planned contrast comparing the model to each human subgroup used the same framework. Accuracy is reported per group; macro‐averaged model metrics are reported across classes. Primary comparison among human groups used one‐way ANOVA with Tukey HSD; planned contrast comparing the model to each human subgroup used the same framework. Accuracy is reported per group; macro‐averaged model metrics are reported across classes.

## RESULTS

### Comparative evaluation of YOLO architectures

Table 1 summarizes the comparative performance of YOLO architectures (v7–v12). YOLOv11x achieved the highest mAP@50–95 (0.915), followed closely by YOLOv12x (0.905). YOLOv12x was selected for its balanced accuracy–speed trade‐off.

### Model performance analysis

**FIGURE 1:**
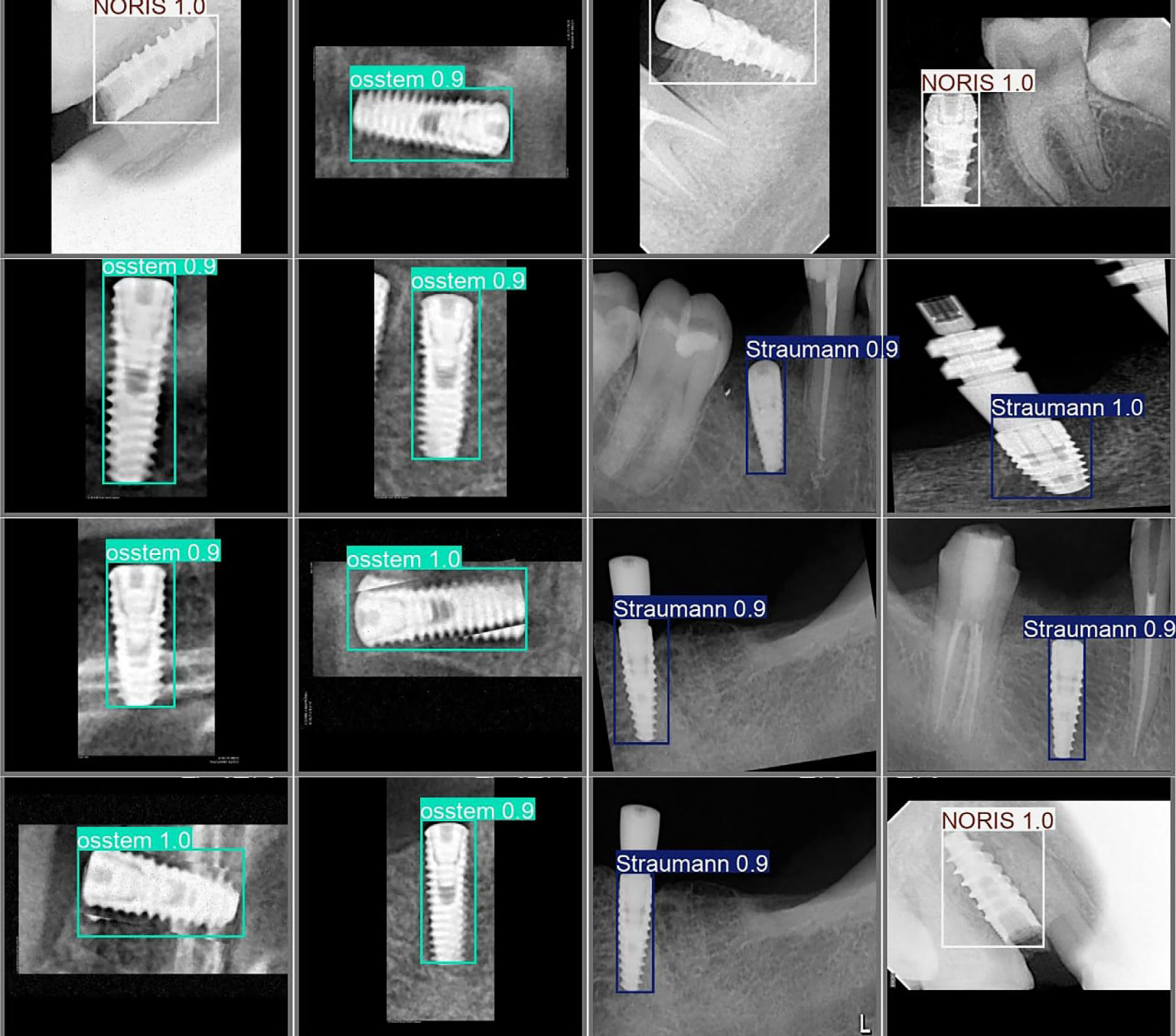
YOLOv12x model accurately detecting and localizing dental implant brands from radiographic images, using bounding boxes and brand labels. Color‐coded annotations correspond to OSSTEM, Straumann, and Noris implants. The model demonstrates high precision and reliability, demonstrating its effectiveness in automated dental implant recognition, aiding in clinical decision‐making, and streamlining prosthodontic workflows.

**FIGURE 2:**
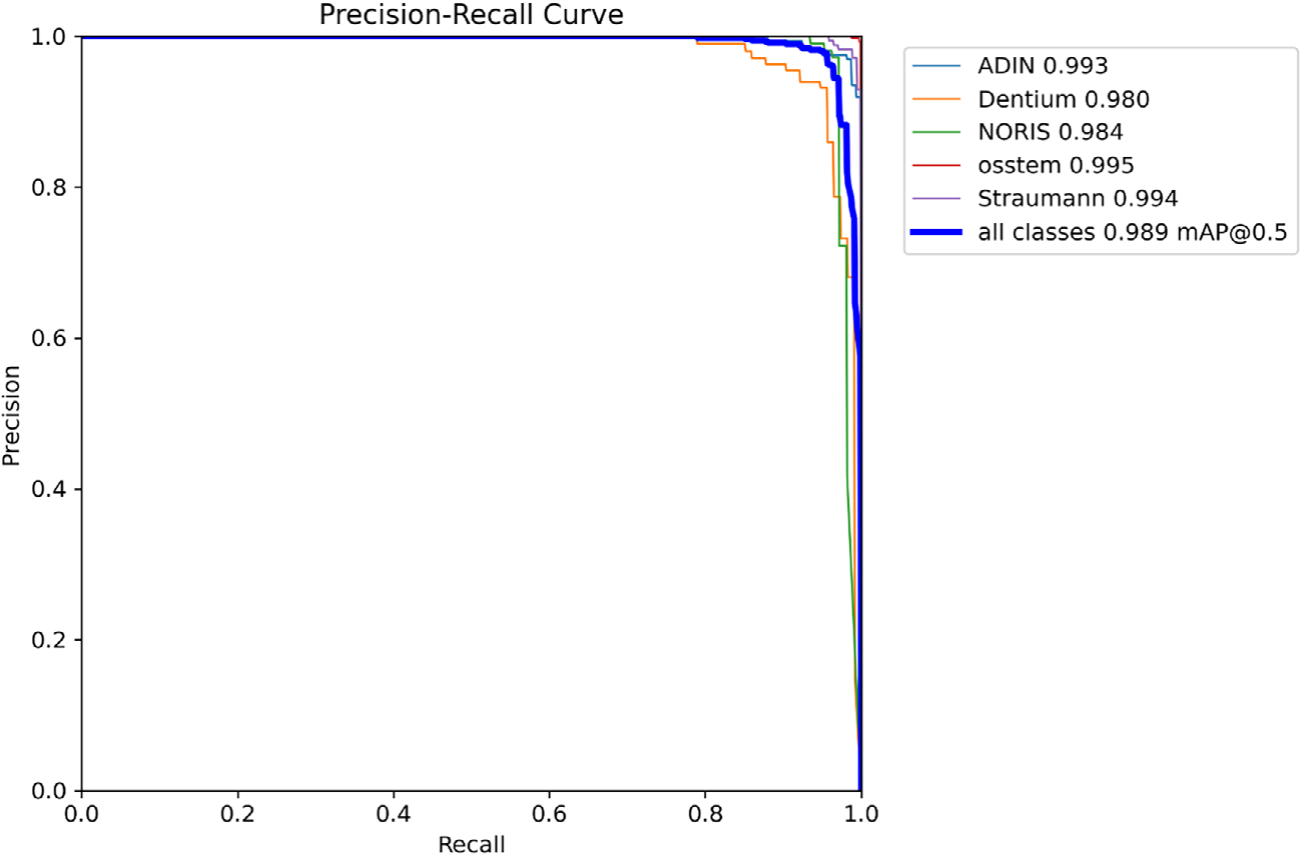
Precision‐recall (PR) curve illustrating the performance of the AI model in detecting and identifying different dental implant brands in radiographic images. The curve shows the balance between precision (the proportion of correct detections) and recall (the proportion of actual implants accurately detected by the model). Each colored line represents a different implant brand (Adin, Dentium, Noris, OSSTEM, or Straumann), whereas the thick blue line represents the overall performance across all brands. The model achieved an average precision of 98.9%, demonstrating its high reliability in accurately detecting and classifying implants. The steep and consistent curves indicate minimal errors, making it a valuable tool for prosthodontists and radiologists to efficiently and accurately identify implants.

**FIGURE 3:**
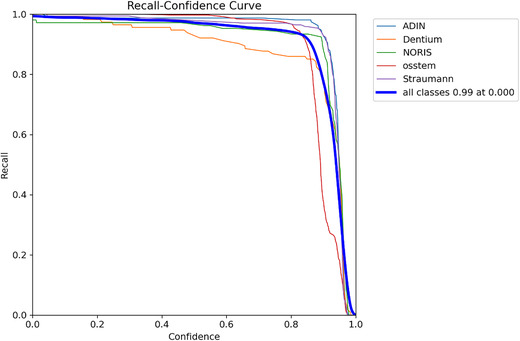
Recall confidence curve used for evaluating how the detection performance of the AI model changes as the confidence thresholds increase. The recall (*y*‐axis) represents the proportion of actual implants successfully identified, whereas the confidence (*x*‐axis) shows the model's certainty in its predictions. Each colored line corresponds to a different implant brand (Adin, Dentium, Noris, OSSTEM, or Straumann), whereas the thick blue line represents the overall model performance. The model maintained high recall values (close to 1.0) across all brands, demonstrating its ability to detect nearly all implants, even at confidence levels. As confidence increases, recall slightly decreases, particularly for brands like Dentium and OSSTEM, indicating that the model becomes more selective. This suggests that at higher confidence levels, the AI makes fewer false detections but may miss some implants. These results confirm that the model provides reliable implant detection while allowing users to adjust the confidence settings based on clinical needs.

**FIGURE 4:**
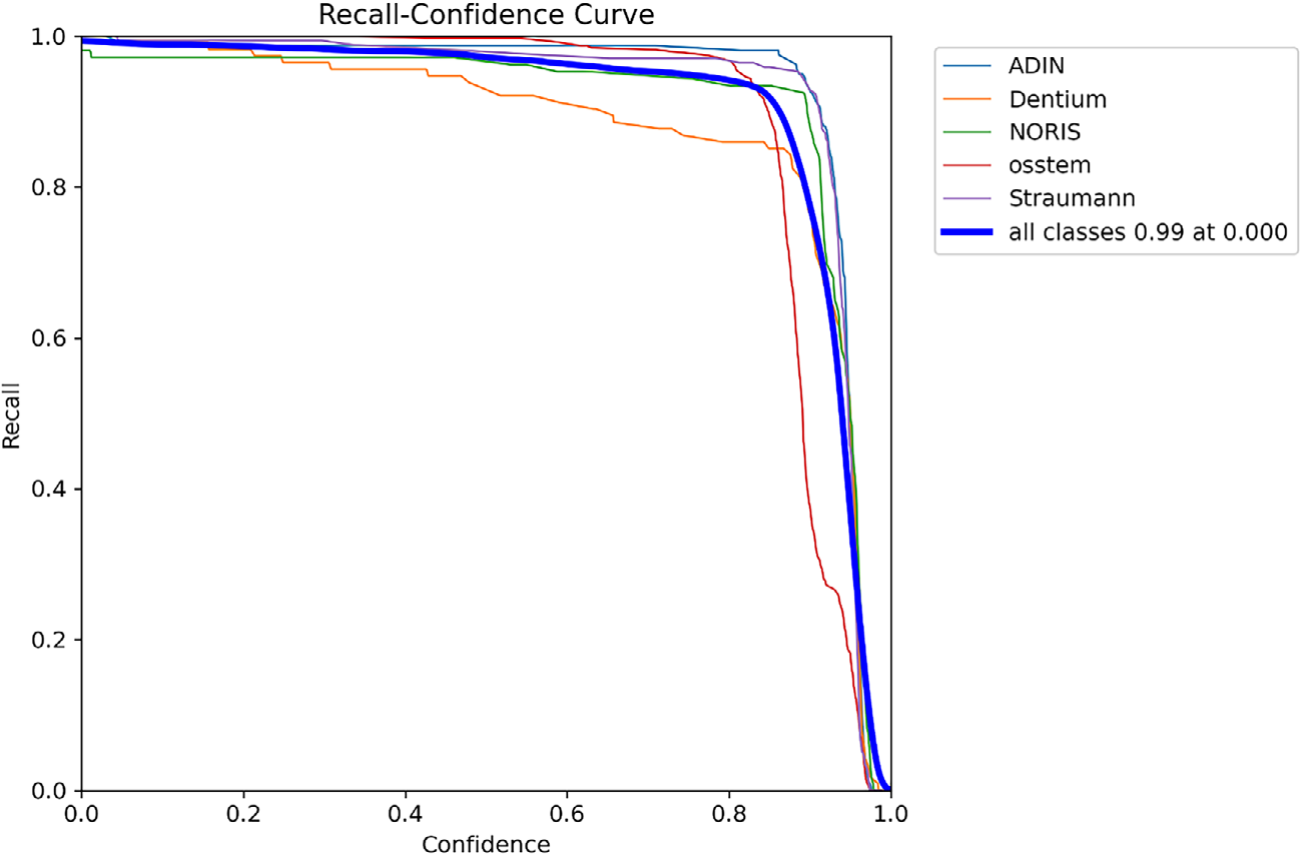
F1‐confidence curve used for assessing the overall performance of the AI model in detecting and classifying dental implants at different confidence levels. The F1‐score (*y*‐axis) represents the balance between precision and recall, whereas the confidence threshold (*x*‐axis) indicates the level of certainty the model requires before making a detection. Each colored line represents a specific implant brand (Adin, Dentium, Noris, OSSTEM, or Straumann), whereas the thick blue line represents the overall model performance across all brands. The model achieved a high F1‐score of 0.97, demonstrating a strong balance between accuracy and sensitivity. As confidence increases, the F1‐score remains high for most brands. However, Dentium and OSSTEM show a slight drop, suggesting that the model becomes more selective and may miss some implants at higher confidence levels. These results confirm that the AI system provides reliable implant detection with minimal errors, making it a valuable tool for clinical applications in prosthodontics and radiology.

**FIGURE 5:**
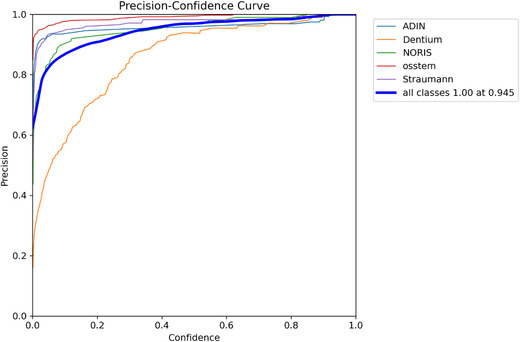
Precision–confidence curve used for evaluating how the accuracy of the AI model in identifying dental implants improves as confidence increases. The precision (*y*‐axis) reflects the proportion of correctly classified implants among all detected implants, whereas the confidence (*x*‐axis) represents the model's certainty threshold for making a prediction. Each colored line corresponds to a specific implant brand (Adin, Dentium, Noris, OSSTEM, and Straumann), whereas the thick blue line represents the overall model performance. The model achieved perfect precision (1.00) for all classes at a confidence threshold of 0.945, indicating that when the model was highly confident, it made almost no mistakes. However, at lower confidence levels, precision initially varied across brands, with Dentium initially showing lower precision before improvement. These results confirm that the AI system provides highly accurate implant detection, allowing dental professionals to adjust the confidence threshold based on clinical requirements to balance accuracy and sensitivity.

**FIGURE 6:**
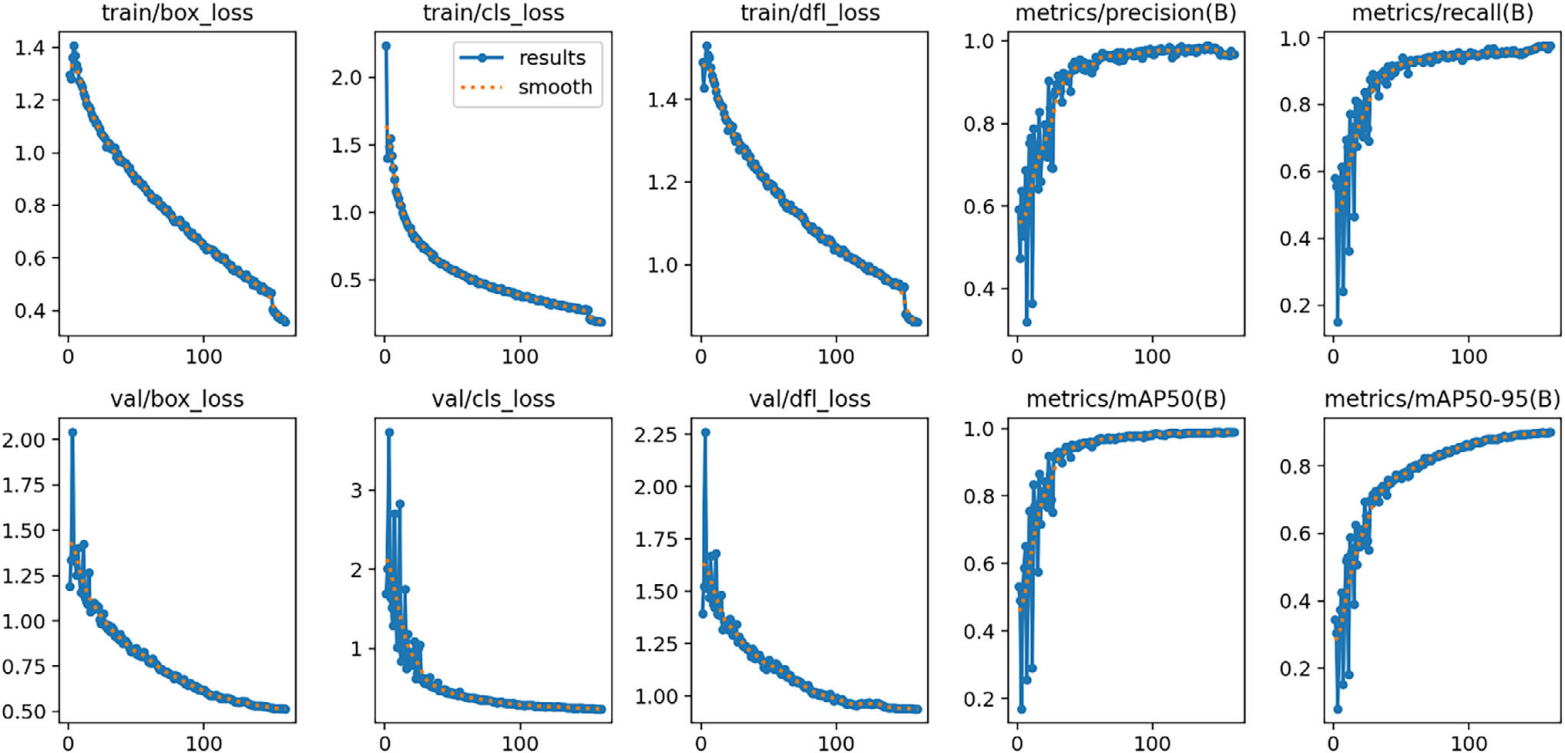
The top row displays the training metrics, whereas the bottom row shows the validation metrics.

The model demonstrated exceptional performance, achieving an overall mAP@50 of 0.989 (98.9%), an overall mAP@50–95 of 0.900 (90.0%), a precision of 0.969 (96.9%), and a recall of 0.977 (97.7%) (Table 2 and Figures 1, 2, 3, 4, 5, 6). The precision‐recall curve for the model is shown in Figure 2, whereas the recall confidence curve is shown in Figure 3. The F1‐confidence curve is depicted in Figure 4, whereas the precision‐confidence curve is shown in Figure 5. The image classification performance of each implant system tested in this study is detailed in Figure 6 and Table 2.

**TABLE 2:** Per class performance.

**Class**	**Images**	**Instances**	**Precision**	**Recall**	**mAP@50**	**mAP@50–95**
Adin	114	159	0.958	0.987	0.993	0.915
Dentium	72	114	0.937	0.947	0.980	0.877
Noris	72	106	0.972	0.972	0.984	0.936
OSSTEM	184	388	0.993	0.997	0.995	0.856
Straumann	142	170	0.982	0.982	0.994	0.916

**FIGURE 7:**
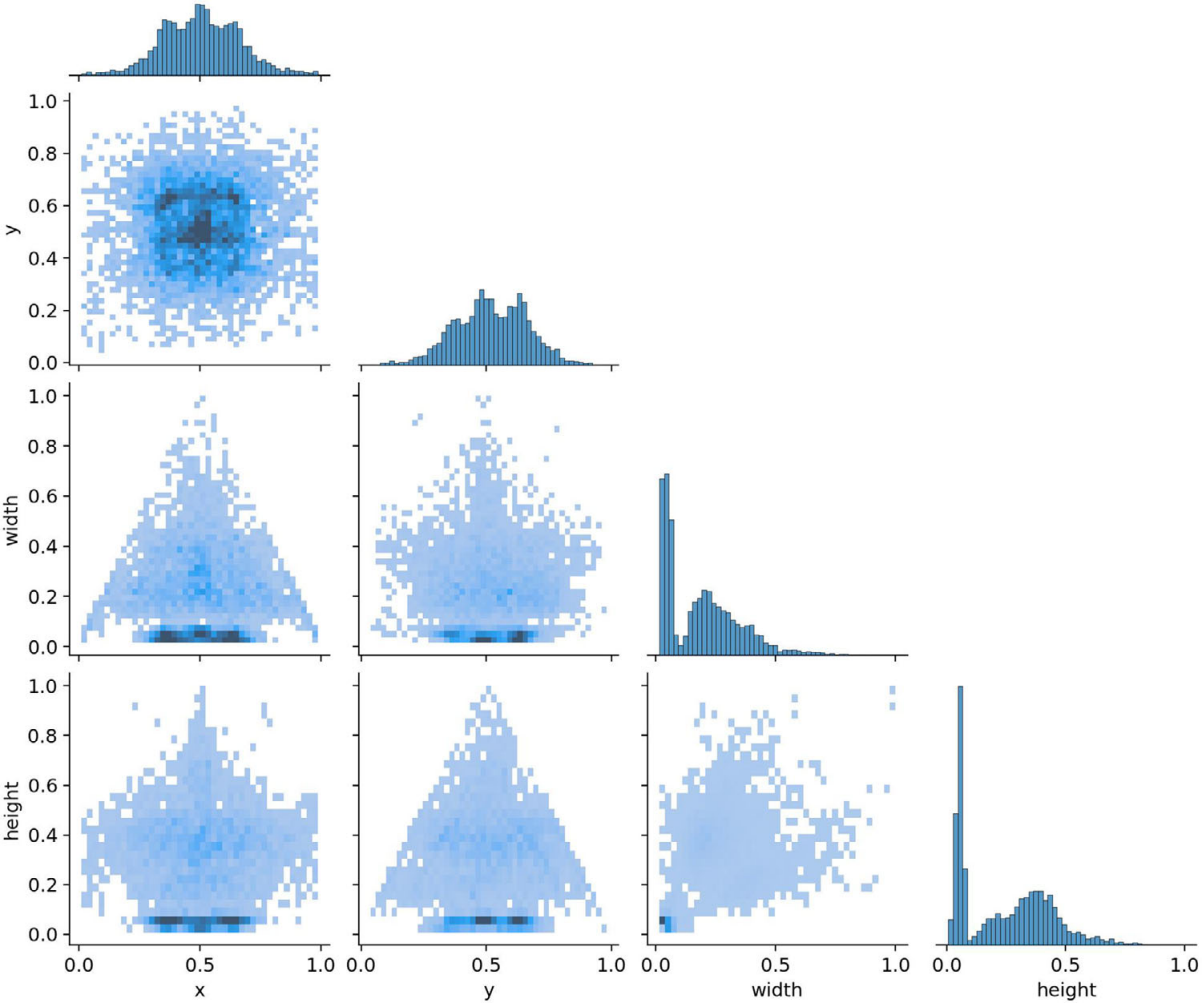
A pair plot (scatter matrix) illustrating the distribution and relationships between the different properties of the detected bounding boxes in the dental implant detection model. Each axis represents a normalized value between 0 and 1, indicating that all values are relative to the image dimensions.

In Figure 7, the diagonal histograms (at the top of each column) show the distribution of individual variables: *x* (horizontal position of the bounding box center), *y* (vertical position of the bounding box center), width (bounding box width), and height (bounding box height). Peaks near (0.5, 0.5) in the *x* and *y* directions suggest that most of the detected implants were located near the center of the image. The width and height distributions show a concentration of smaller bounding boxes. The off‐diagonal scatter plots were used to visualize the relationships between the different bounding box attributes (Figure 7). The *x* versus *y* plot indicates that most bounding boxes are centered around the middle of the image.

**FIGURE 8:**
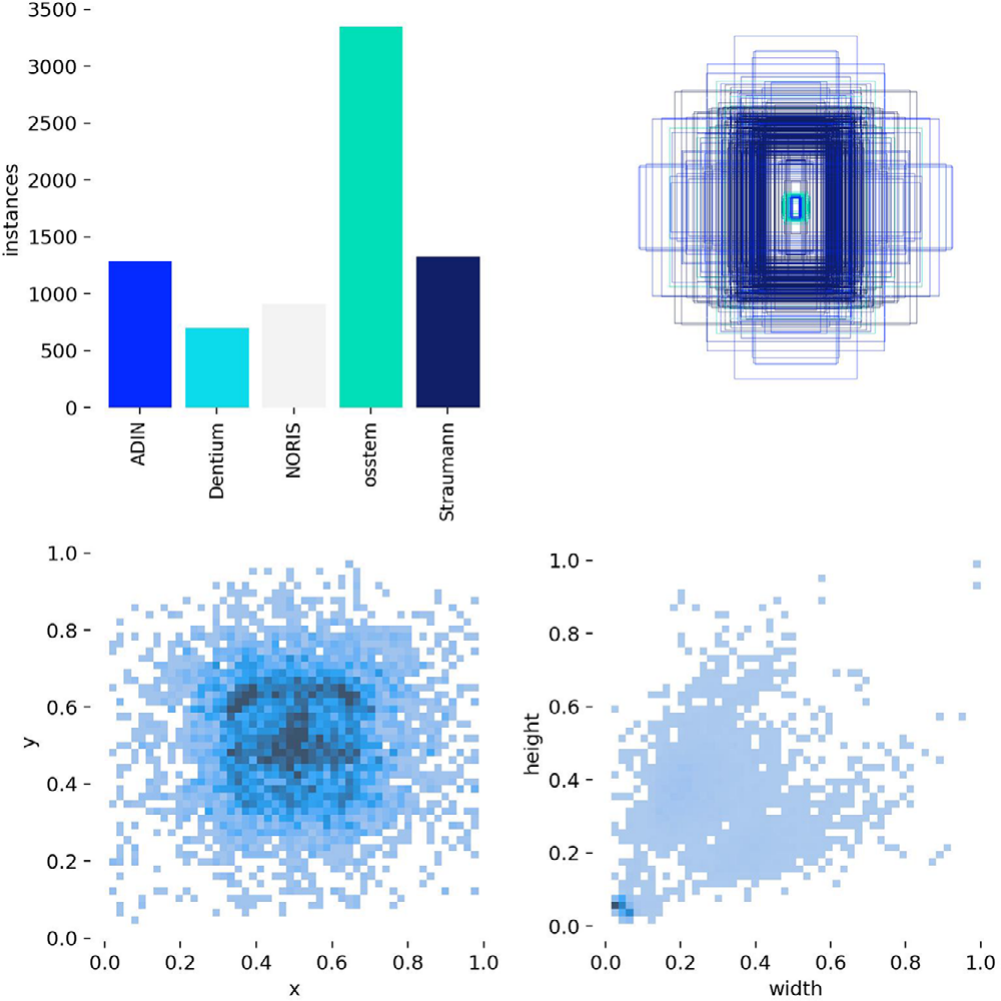
An in‐depth analysis of the distribution of the detected dental implants in terms of class frequencies, bounding box placements, and size distributions. Each subplot provides insight into how the AI model identifies and localizes implants in the x‐ray images.

The class distribution displays the number of detected instances for each dental implant brand (Adin, Dentium, NORIS, OSSTEM, and Straumann) (Figure 8). The OSSTEM implants had the highest number of instances, suggesting a larger dataset for this brand. Dentium had the lowest count, which could indicate fewer training samples, potentially affecting model performance for this brand. A well‐balanced dataset is preferred, but variations in class frequency may influence classification accuracy. In Figure 8, the Bounding Box Overlays show the spatial distribution of the detected bounding boxes overlaid on a normalized grid (scale of 0 to 1). The densely packed central region suggests that most implants were detected near the center of the image. Lighter outer regions indicate fewer detections near the edges. This discrepancy may be due to image framing or inherent dataset bias. In addition, Figure 8 shows the center distribution of the bounding box in the bottom left scatter plot (*x* vs. *y*), representing the normalized center positions of all detected bounding boxes. The dark central clustering confirmed that most implants were positioned near the center of the image. The spread toward the edges suggests that some implants appeared in the peripheral regions, although less frequently. The bottom‐right scatter plot in Figure 8 illustrates the bounding box size distribution (width vs. height), showing the relationship between the bounding box width and height. Most bounding boxes were small (low width and height values), suggesting that implants are compact structures in x‐ray images. Outliers with larger bounding boxes could indicate cases with multiple implants or variations in the imaging scale.

**FIGURE 9:**
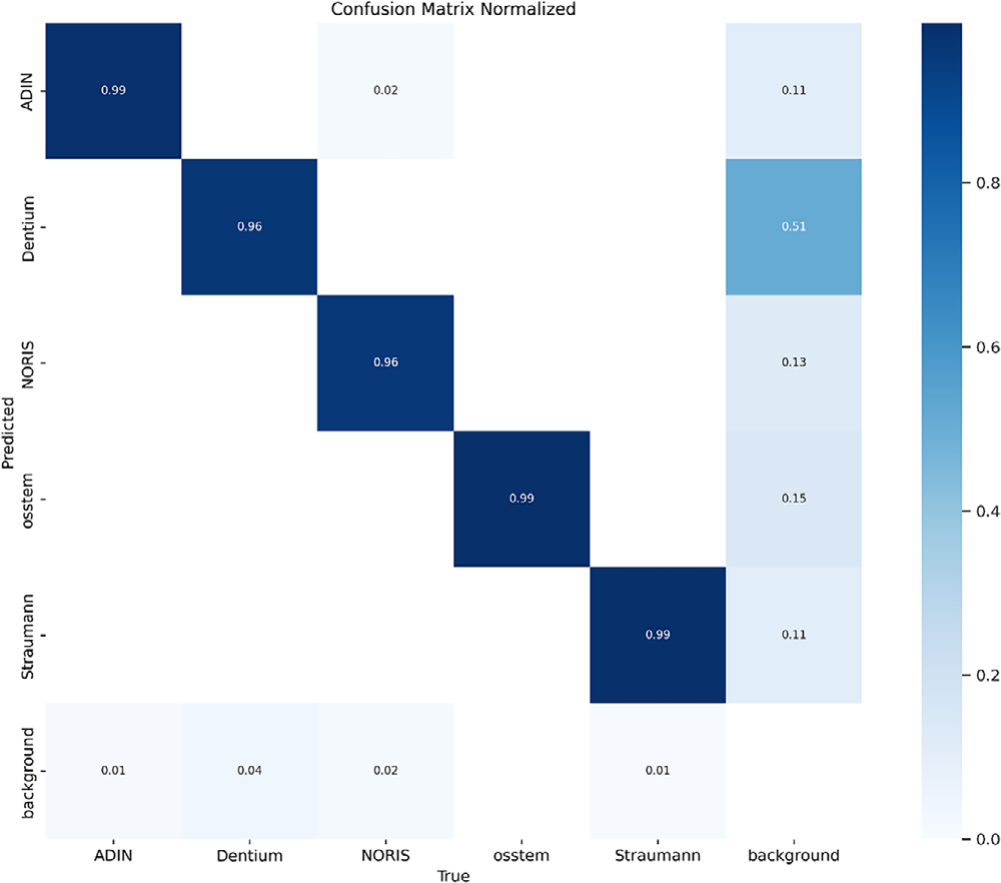
Normalized confusion matrix evaluating the classification performance of the AI model for dental implant brands, displaying the proportions of correct and incorrect predictions for each class.

The diagonal values (dark blue) indicate high accuracy, with 99% correct classifications for Adin, OSSTEM, and Straumann. However, Dentium and NORIS exhibited slightly lower accuracies (96%), suggesting some misclassification (Figure 9). Overall, the model performed well; however, improvements may be required to reduce background misclassification, particularly for Dentium implants.

**FIGURE 10:**
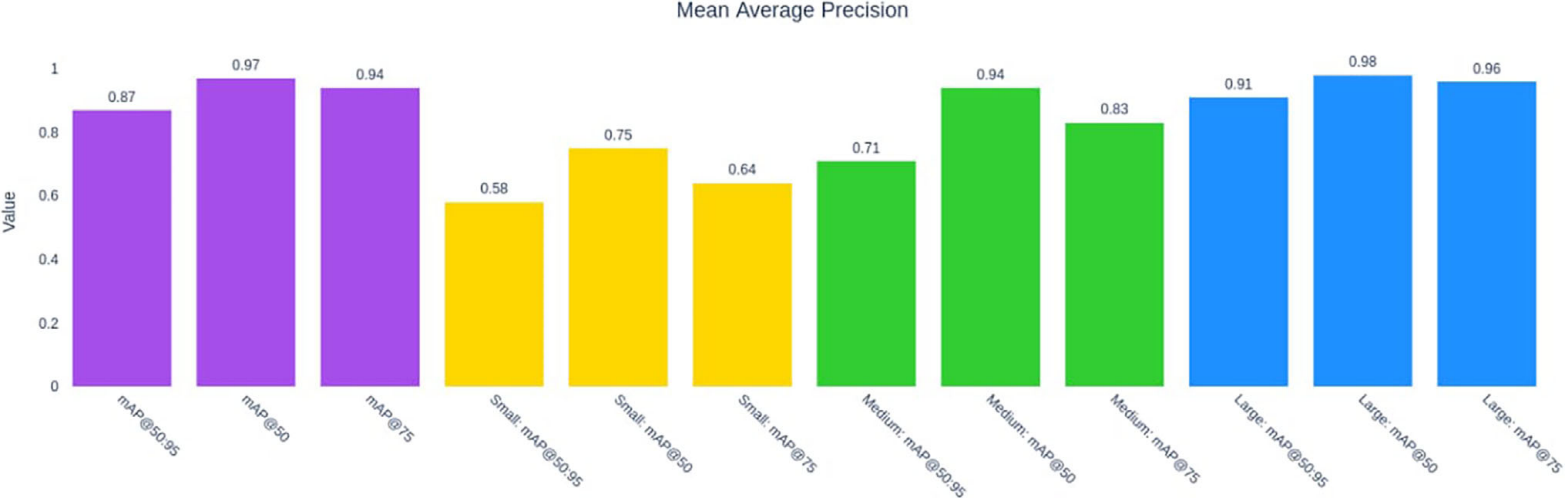
Bar chart illustrating the mean average precision (mAP) of the AI model at different Intersection over Union (IoU) thresholds and for different object sizes (small, medium, and large). mAP is a key metric in object detection, assessing the model's ability to detect and localize implants. Higher values indicate better performance. The color‐coded representation is as follows: overall mAP scores (purple bars), performance on small implants (yellow bars), performance on medium implants (green bars), and performance on large implants (blue bars).

Figure 10 displays the overall mAP scores. mAP(50:95) = 0.87 indicates the average mAP across Intersection over Union (IoU) thresholds from 50% to 95%, reflecting stricter evaluation criteria. mAP@50 = 0.97 measures detection performance at 50% IoU, signifying high reliability in identifying implants. mAP@75 = 0.94, which requires 75% overlap, demonstrates that the model performs well even under stricter localization demands. Performance on small implants showed lower scores (0.58–0.75), suggesting that the model faces challenges in detecting smaller implants, likely because their finer details are more difficult to distinguish in x‐ray images. Performance on medium implants showed higher scores (0.71–0.94) compared with small implants but slightly lower than large implants, indicating moderate detection accuracy. Performance on large implants showed the highest scores (0.91–0.98), indicating that the model detects and localizes larger implants with high confidence and precision.

### Comparative analysis between the AI model and human participants

**FIGURE 11:**
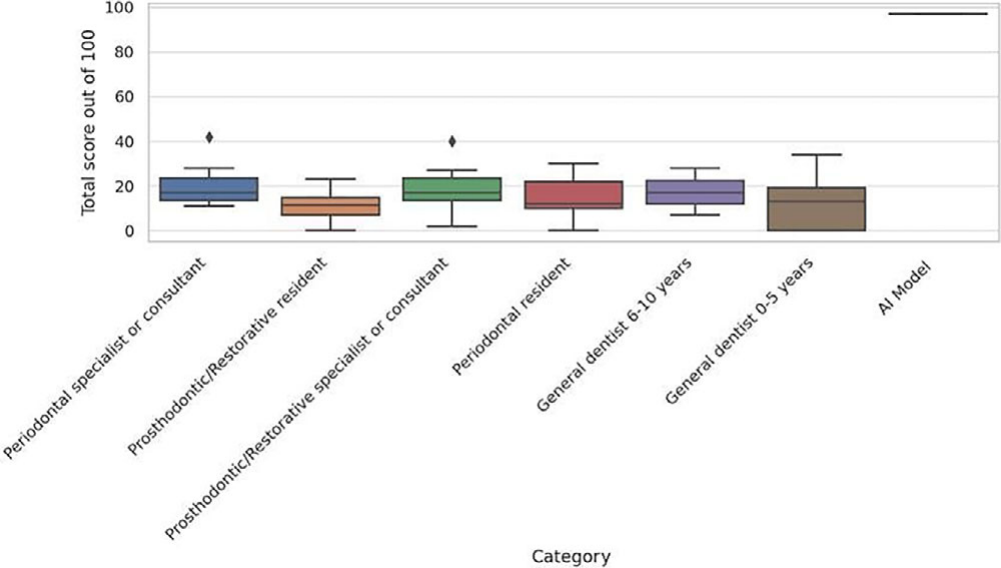
Total score distribution by human group.

A total of 52 human participants from six clinical categories participated in the implant brand identification task. Each participant evaluated 100 radiographic images, yielding 5200 responses. The average score was 31.06 ± 12.99 (mean ± standard deviation) out of 100, with scores ranging from 3 to 61 (Table 3). The proportion of “I don't know” responses was notably high, accounting for 47.54% (*n* = 2472) of the total responses. A breakdown of scores by clinical group showed no significant differences in performance among consultant‐level clinicians, residents, and general practitioners. Figure 11 presents the distribution of total scores across the six subgroups. The mean scores for each group are as follows: General dentist (0–5years): 22.4 ± 7.4, general dentist (> 5 years): 27.0 ± 10.3, periodontics resident: 26.1 ± 8.2, prosthodontics/restorative resident: 25.6 ± 10.6, periodontal consultant: 30.6 ± 13.0, and prosthodontic/restorative consultant: 29.6 ± 12.9. By contrast, the AI model achieved an accuracy of 95%, correctly identifying the implant brand in 95% of the cases in the test dataset (Table 3).

**TABLE 3:** Descriptive statistics of the total scores for each group, including the mean, median, standard deviation, interquartile range, minimum and maximum scores, and score range.

Category	Count	Mean	Median	SD	Min	Max	IQR	Range
AI model	10	97.0	97.0	0.0	97	97	0.0	0
General dentist: 0–5 years	8	12.38	13.0	12.42	0	34	19.25	34
General dentist: 6–10 years	3	17.33	17.0	10.5	7	28	10.5	21
Periodontal resident	7	15.14	12.0	10.65	0	30	12.0	30
Periodontal specialist or consultant	7	20.57	17.0	11.0	11	42	10.0	31
Prosthodontic/restorative resident	10	11.1	11.5	7.34	0	23	7.75	23
Prosthodontic/restorative specialist or consultant	7	19.0	17.0	12.0	2	40	10.0	38

Abbreviations: IQR, interquartile range; Max, maximum; Min, minimum; SD, standard deviation.

### Implant‐level accuracy and confusion patterns

**FIGURE 12:**
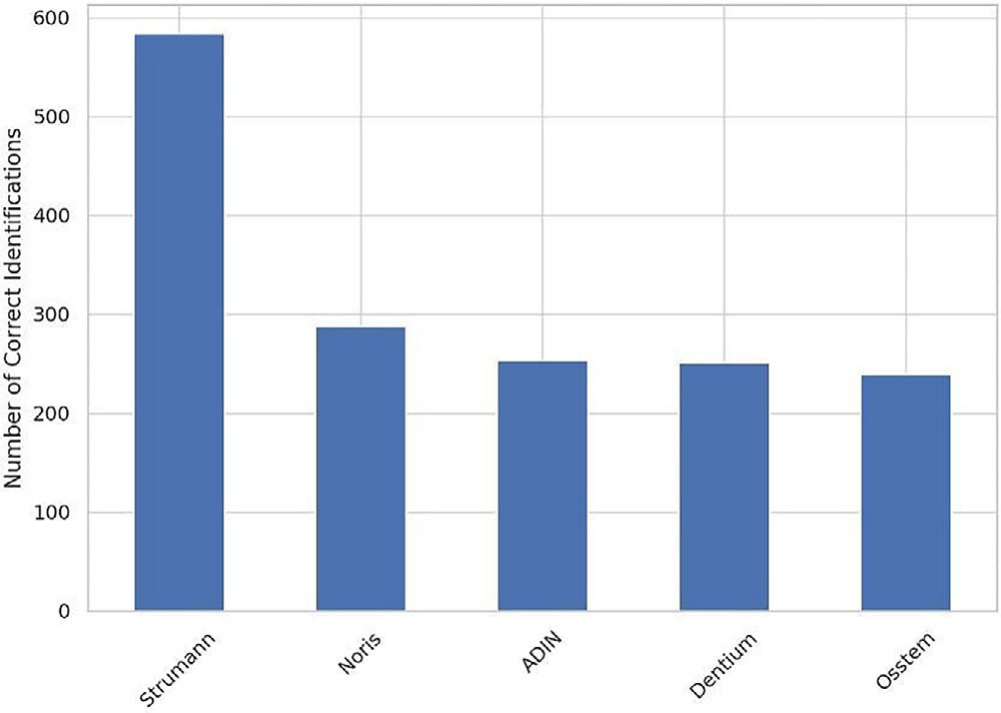
Number of correct identifications per implant brand.

**FIGURE 13:**
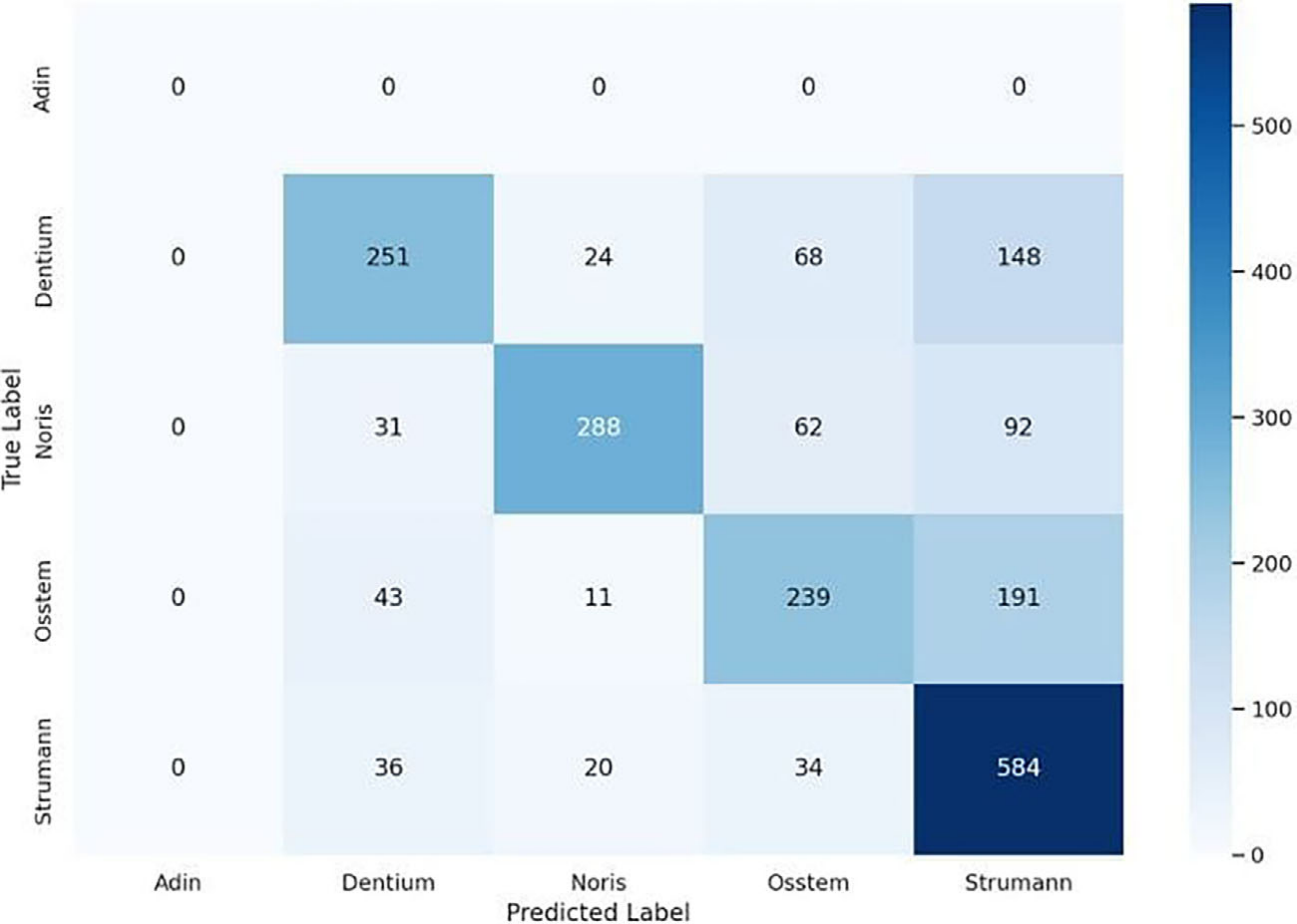
Confusion matrix highlighting the patterns of misidentification.

The implants most frequently identified correctly by human participants were Straumann (*n* = 584), followed by Noris (*n* = 288) and Dentium (*n* = 251). Adin was the least correctly identified brand, with 0 correct identifications. Figure 12 shows the number of correct identifications for each implant brand. The confusion matrix (Figure 13) highlights the patterns of misidentification. Dentium was often confused with Straumann (*n* = 148) or OSSTEM (*n* = 68). Osstem was misidentified as Straumann in 191 instances. Noris was also confused with Straumann (*n* = 92). These results suggest a tendency among human evaluators to overselect familiar or visually similar brands (especially Straumann) when uncertain.

### Inferential statistical analysis

To assess the differences in diagnostic accuracy among the participant groups, a one‐way analysis of variance (ANOVA) was conducted. A Tukey's honestly significant difference (HSD) post hoc analysis was used to identify specific group differences (Table 4). The AI model was included in the initial comparisons, with a separate analysis conducted for human groups only. The one‐way ANOVA was conducted to assess the differences in accuracy across the human groups. The results indicated a significant difference between the groups (*F*(5, 46) = 100.56, *p* < 0.001) (Table 3).

**TABLE 4:** Tukey HSD post hoc test (human groups alone).

Group 1	Group 2	Mean difference	*p*‐adj	Lower	Upper	Reject
AI model	General dentist: 0–5 years	−84.625	0.0	−98.52	−70.73	True
AI model	General dentist: 6–10 years	−79.6667	0.0	−98.9498	−60.3835	True
AI model	Periodontal resident	−81.8571	0.0	−96.293	−67.4213	True
AI model	Periodontal specialist or consultant	−76.4286	0.0	−90.8644	−61.9927	True
AI model	Prosthodontic/restorative resident	−85.9	0.0	−99.0003	−72.7997	True
AI model	Prosthodontic/restorative specialist or consultant	−78.0	0.0	−92.4359	−63.5641	True
General dentist: 0–5 years	General dentist: 6–10 years	4.9583	0.9866	−14.8733	24.7899	False
General dentist: 0–5 years	Periodontal resident	2.7679	0.9975	−12.3928	17.9285	False
General dentist: 0–5 years	Periodontal specialist or consultant	8.1964	0.64	−6.9643	23.3571	False
General dentist: 0–5 years	Prosthodontic/restorative resident	−1.275	1.0	−15.17	12.62	False
General dentist: 0–5 years	Prosthodontic/restorative specialist or consultant	6.625	0.8253	−8.5357	21.7857	False
General dentist: 6–10 years	Periodontal resident	−2.1905	0.9999	−22.4047	18.0238	False
General dentist: 6–10 years	Periodontal specialist or consultant	3.2381	0.9988	−16.9761	23.4523	False
General dentist: 6–10 years	Prosthodontic/restorative resident	−6.2333	0.952	−25.5165	13.0498	False
General dentist: 6–10 years	Prosthodontic/restorative specialist or consultant	1.6667	1.0	−18.5476	21.8809	False
Periodontal resident	Periodontal specialist or consultant	5.4286	0.9337	−10.2293	21.0865	False
Periodontal resident	Prosthodontic/restorative resident	−4.0429	0.9761	−18.4787	10.393	False
Periodontal resident	Prosthodontic/restorative specialist or consultant	3.8571	0.9876	−11.8007	19.515	False
Periodontal specialist or consultant	Prosthodontic/restorative resident	−9.4714	0.4142	−23.9073	4.9644	False
Periodontal specialist or consultant	Prosthodontic/restorative specialist or consultant	−1.5714	0.9999	−17.2293	14.0865	False
Prosthodontic/restorative resident	Prosthodontic/restorative specialist or consultant	7.9	0.6269	−6.5359	22.3359	False

Abbreviations: HSD, honestly significant difference; *p*‐adj, adjusted *p* value.

Across 52 clinicians in six subgroups, the model's accuracy exceeded each subgroup (ANOVA F[5, 46] = 100.56; Tukey HSD *p* < 0.001 for all pairwise comparisons vs. model). In the pre‐specified core 16 subgroup, mean accuracy remained significantly below the model (Tukey HSD *p* < 0.001); no significant differences emerged among the four professional levels. Model macro‐averaged metrics across classes were precision = 0.968, recall = 0.977, mAP@50 = 0.989, and mAP@50–95 = 0.900. The AI model significantly outperformed all human groups (*p* < 0.001 for all comparisons). No significant differences were found among the human groups (all *p* > 0.05), suggesting that years of experience and specialization did not lead to significantly higher performance. These findings highlight that, although AI substantially outperforms human participants in implant identification tasks, the performance of human evaluators remains consistently low, regardless of experience or specialization.

## DISCUSSION

This study presents the development and evaluation of a DL model for the automated detection and classification of dental implants. The model was trained using a dataset of five dental implant images. After data preprocessing and class filtering, the model achieved outstanding performance, with an overall mAP@50 of 0.989 and mAP@50–95 of 0.9 across implant brands. These results demonstrate the model's exceptional performance, highlighting its potential for clinical applications in prosthodontics, particularly in implant identification and treatment planning.

YOLOv12x was selected for this study due to its advanced architecture and exceptional performance in object detection tasks, particularly within the field of medical image analysis. This model incorporates several innovative features that enhance its suitability for dental implant detection. First, YOLOv12x employs a novel self‐attention approach that efficiently processes large receptive fields by dividing feature maps into equally sized regions (typically four), either horizontally or vertically. This approach preserves a large effective receptive field while significantly reducing the computational cost compared with standard self‐attention methods, making it particularly effective for detecting implants of varying sizes in radiographic images. Second, the model incorporates an improved feature aggregation module (residual efficient layer aggregation network), which includes block‐level residual connections and scaling, along with a redesigned bottleneck‐like feature aggregation structure. These enhancements address optimization challenges in attention‐centric models, enabling better feature extraction from complex dental radiographic images. Third, YOLOv12x streamlines attention mechanisms specifically for efficiency and compatibility with the YOLO framework. It leverages FlashAttention implementation to minimize memory overhead, eliminate traditional positional encoding for faster processing, and balance the MLP ratios (reduced from 4 to 1.2–2) to optimize computation between the attention and feed‐forward layers. Additionally, the depth of the stacked blocks was reduced to improve optimization and strategically utilize convolution operations for computational efficiency. Furthermore, the integration of a 7 × 7 separable convolution (“position perceiver”) that implicitly encodes positional information is crucial for the precise localization of dental implants. These architectural innovations make YOLOv12x particularly well‐suited for the challenging task of dental implant detection, where precise localization and classification are essential for clinical applications. Benchmarking against the DF‐DETR transformer baseline (mAP@50–95 = 0.878) demonstrated that YOLOv12x retained superior fine‐grained localization and inference efficiency, confirming its suitability for high‐volume dental image analysis.

All classes achieved mAP@50 values exceeding 0.98 (98%), demonstrating exceptional detection ability. The OSSTEM class achieved the highest performance, with a precision of 0.993 and a recall of 0.997. The mAP@50–95 scores (ranging from 0.856 to 0.936) indicated robust detection performance across various IoU thresholds. Dentium showed slightly lower metrics compared with other classes but still maintained excellent performance. The developed model represents a significant advancement in prosthodontic practice in terms of efficiency, accuracy, scalability, and potential integration into digital radiology systems. It enables the rapid identification of implant systems without manual referencing with high precision, whereas its recall capabilities minimize identification errors. This framework can be extended to include additional implant brands and demonstrates the potential for integration into digital radiography systems.

In Figure 7, the *x* versus width and *y* versus height plots exhibit a triangular distribution, indicating that the bounding boxes tend to be smaller near the edges and larger toward the center. The width versus height plot indicated that most bounding boxes maintain a consistent aspect ratio, likely due to the standard shape of the implants in the x‐ray images. The *x* versus *y* plot confirms that most bounding boxes are centered within the images. The AI model predominantly detects implants with relatively small bounding boxes, suggesting it is optimized for identifying fine details. Additionally, a concentration of specific aspect ratios suggests that the implants exhibit consistent proportions across images. A few large bounding boxes are present, potentially indicating that most implants covered only a small portion of the x‐ray images. These findings validate the model's bounding box predictions, confirming that the detected implants are well distributed and positioned logically within the images. Overall, these results suggest that the AI system can accurately detect and localize dental implants, making it a valuable tool for clinical applications.

OSSTEM implants dominate the dataset, which may lead to better model performance for this brand and potentially lower accuracy for underrepresented brands, such as Dentium. This confirms that the AI model accurately detects and localizes dental implants, with most detections occurring in the center of the images. However, the imbalance in the class distribution suggests that performance may vary across brands. These insights highlight the importance of improving dataset balance to optimize detection accuracy for all implant types.

Overall, the model performed exceptionally well (mAP@50 = 0.97, mAP@75 = 0.94), demonstrating its ability to accurately detect and classify dental implants (Figure 10). The detection of small implants proved challenging (mAP@50–95 = 0.58), likely due to their size, low contrast, or occlusions in x‐ray images. Medium and large implants were detected with high accuracy, indicating the model's robustness in most cases. Although the model excels at detecting medium and large implants, improvements are needed for detecting small implants. Enhancing resolution and feature extraction could improve small implant detection. Balanced dataset augmentation may help address this performance gap. Overall, this analysis confirms the AI model's high reliability for dental implant detection, with only minor improvements required for smaller implants.

Among recently published studies evaluating the accuracy of AI for detecting and classifying dental implants,^7^, ^8^, ^9^, ^12^, ^13^, ^14^, ^15^, ^16^, ^17^, ^18^, ^19^, ^20^, ^21^ the overall accuracy of AI models ranged from 67% to 98.5%, depending on the model architecture and sample size. Most studies have demonstrated mean accuracy levels exceeding 90%. Our study achieved a higher accuracy (98%), surpassing the previously published results. This superior performance may be attributed to the adoption of the recently introduced YOLO12v model and the use of higher‐resolution images. A cross‐version benchmarking of YOLO architectures (v7–v12) demonstrated consistent advancement in precision and recall. Although YOLOv11x achieved the highest mAP@50–95 (0.915), YOLOv12x provided near‐equivalent accuracy with improved inference speed, validating its measurable advantage over previous versions (Table 1).

Four studies^13^, ^16^, ^20^, ^21^ compared the accuracy of AI tools in implant identification with that of trained dental professionals. All studies reported that the accuracy of the DL algorithm was significantly better than that of dentists. Park et al.^21^ reported an AI accuracy of 82.3%, whereas dentist accuracy ranged from 16.8% (among nonspecialists) to 43.3% (among specialists in implantology). Lee et al.^16^ reported mean accuracies of 80.56% for an automated DL algorithm, 63.13% for participants without AI assistance, and 78.88% for those with AI assistance. These results highlight the significant improvement in classification accuracy when DL support was provided. In another study, Lee et al.^20^ reported similar findings. Similarly, Lee et al.^13^ reported accuracy rates of 97.1% for DLs and 92.5% for periodontists.

The AI model significantly outperformed all human groups, likely due to its exposure to a large and consistent training dataset. Interestingly, increased clinical experience or specialization did not correlate with higher diagnostic performance, highlighting inherent limitations in human radiographic pattern recognition when distinguishing between similar implant systems. The high rate of “I don't know” responses further underscores the diagnostic uncertainty clinicians face in this task. By contrast, the AI demonstrated superior and consistent performance in identifying dental implant brands from radiographic images. Its integration into clinical practice could assist practitioners in cases of uncertainty and enhance diagnostic efficiency.

Despite the excellent performance, several limitations must be acknowledged. First, five major implant brands were included in the training dataset; therefore, expansion to other systems would enhance its clinical utility. Second, the presence of novel implant designs may have reduced the accuracy, as these were not represented in the current training data. Moreover, model performance may be influenced by variations in radiographic quality and image angulation. Finally, performance reflected the underlying class distribution (OSSTEM overrepresented). This study did not include an external validation cohort; generalizability is therefore limited. While per‐class metrics are reported, future studies will alleviate imbalance with class‐weighted loss or targeted data augmentation and resampling. Importantly, this study lacked external validation, and multisite evaluation with different models is planned. Moreover, given the high ‘I don't know’ rate among clinicians, the most promising path may be human–AI collaboration: the model triages images, surfaces a top‐N brand list with confidence, and flags low‐confidence cases for specialist review, aiming to reduce uncertainty and time‐to‐identification. Future studies should assess the usability of AI in real‐world clinical workflows and extend evaluation to other implant systems. Several promising avenues for development include expanding detection capabilities to cover more implant brands and variants, improving performance across diverse radiographic angulations through multi‐angle detection, extending detection to specific implant components (such as abutments and screws), and developing user‐friendly interfaces to facilitate clinical implementation.

## Conclusions

This study successfully developed a high‐performance AI system for dental implant detection and classification with exceptional accuracy across five major implant brands. The YOLOv12x model achieved an overall mAP@50 of 0.989 and an mAP@50–95 of 0.9, demonstrating its potential as a valuable clinical tool for prosthodontic practice. The AI model significantly outperformed all human groups. Further refinement and expansion of the system could significantly impact clinical workflow efficiency and improve treatment planning accuracy in prosthodontic practice. YOLOv12x outperforms earlier versions in overall stability and speed. These findings validate YOLOv12x as a robust, clinically applicable framework capable of precise and real‐time identification of implant systems.

## Data Availability

Data are available upon request.
